# Novel Organoruthenium(II) Complex C1 Selectively Inhibits Butyrylcholinesterase without Side Effects on Neuromuscular Transmission

**DOI:** 10.3390/ijms24032681

**Published:** 2023-01-31

**Authors:** Tomaž Trobec, Monika C. Žužek, Kristina Sepčić, Jerneja Kladnik, Iztok Turel, Robert Frangež

**Affiliations:** 1 Institute of Preclinical Sciences, Veterinary Faculty, University of Ljubljana, Gerbičeva 60, 1000 Ljubljana, Slovenia; 2 Department of Biology, Biotechnical Faculty, University of Ljubljana, Jamnikarjeva 101, 1000 Ljubljana, Slovenia; 3 Department of Chemistry and Biochemistry, Faculty of Chemistry and Chemical Technology, University of Ljubljana, Večna pot 113, 1000 Ljubljana, Slovenia

**Keywords:** organoruthenium(II) complex, acetylcholinesterase, butyrylcholinesterase, mouse neuromuscular system

## Abstract

Enzyme butyrylcholinesterase (BChE) shows increased activity in some brain regions after progression of Alzheimer’s disease and is therefore one of the therapeutic targets for symptomatic treatment of this neurodegenerative disorder. The organoruthenium(II) complex [(η^6^-*p*-cymene)Ru(II)(1-hydroxy-3-methoxypyridine-2(1*H*)-thionato)pta]PF_6_ (**C1**) was designed based on the results of our previous structure–activity studies. Inhibitory activity toward cholinesterase enzymes shows that this complex selectively, competitively, and reversibly inhibits horse serum BChE (hsBChE) with an IC_50_ value of 2.88 µM. When tested at supra-pharmacological concentrations (30, 60, 90, and 120 µM), **C1** had no significant effect on the maximal amplitude of nerve-evoked and directly elicited single-twitch and tetanic contractions. At the highest tested concentration (120 µM), **C1** had no effect on resting membrane potential, but significantly decreased the amplitude of miniature end-plate potentials (MEPP) without reducing their frequency. The same concentration of **C1** had no effect on the amplitude of end-plate potentials (EPP), however it shortened the half-decay time of MEPPs and EPPs. The decrease in the amplitude of MEPPs and shortening of the half-decay time of MEPPs and EPPs suggest a possible weak inhibitory effect on muscle-type nicotinic acetylcholine receptors (nAChR). These combined results show that, when applied at supra-pharmacological concentrations up to 120 µM, **C1** does not importantly affect the physiology of neuromuscular transmission and skeletal muscle contraction.

## 1. Introduction

Ruthenium complexes can interact with various biological targets in different organisms (viruses, bacteria, fungi, parasites, animals, and humans) [1,2,3,4,5,6,7]. In animals and humans, these targets are often enzymes involved in physiological and pathophysiological processes. In humans, this group of enzymes includes cholinesterases (ChEs), lactate dehydrogenase, glutathione S-transferases, protein tyrosine phosphatase, protein kinases, glycogen synthase kinase 3, aldo-keto reductase, thioredoxin reductase, and many others [8,9,10,11,12,13]. It is interesting to note that organoruthenium(II) complex with pyrithione also showed moderate activity against selected enzymes (especially papain-like protease (PLpro)) involved in SARS-CoV-2 entry and replication, though the activity of the corresponding zinc complex was higher [14]. Organoruthenium complexes are very promising not only as anticancer agents but also as agents for symptomatic therapy of neurodegenerative diseases due to their inhibitory properties against both acetycholinesterase (AChE) and butyrylcholinesterase (BChE), as well as amyloid-β (Aβ) aggregation [2,9,15,16,17]. In the last decade, our research group has focused on the development and testing of organoruthenium(II) complexes derived from various precursors (including *p*-cymene and CORM 2), having attached chelating (pyrithione, *β*-diketonates, methoxypyridine, nitrophenantroline) as well as monodentate ligands (Cl^−^, Br^−^, I^−^, 1,3,5-triaza-7-phosphaadamantane—**pta**). In vitro biochemical characterization showed that some organoruthenium(II) complexes were non-selective inhibitors of ChE enzymes (e.g., organoruthenium(II) pyrithione complex with Cl^−^, Br^−^, and I^−^ ligands), while some others selectively inhibited only BChE (e.g., organoruthenium(II) pyrithione complex with **pta** ligand) [2,18]. The cholinesterase enzyme family includes two members: AChE and BChE. AChE is a substrate-specific ChE and mainly catalyzes the hydrolysis of acetylcholine, whereas BChE has a much broader substrate specificity and hydrolyzes a variety of choline and non-choline esters [19,20,21]. In mammals, AChE is found in cholinergic synapses of the central, peripheral, and autonomic nervous systems, neuromuscular junctions, and erythrocytes, whereas BChE is found especially in plasma, liver, and also in neuromuscular junctions and some regions in the brain (glial cells, endothelial cells, and neurons) [21,22,23]. In addition to its detoxifying role, BChE retains some function in the process of neurotransmission [22]. For example, BChE can play an important role as a substitute for AChE when AChE activity is suppressed or absent. In the brain, BChE, unlike AChE, is mainly located outside the synaptic cleft [19,22].

Cholinesterase inhibitors slow down or completely inhibit the activity of ChE and subsequently the degradation of acetylcholine to acetate and choline. This prolongs the availability and duration of action of acetylcholine in the synaptic cleft [23,24]. Until today, the Food and Drug Administration (FDA) and European Medicines Agency (EMA) have approved only four ChE inhibitors: tacrine, donepezil, rivastigmine, and galantamine [25]. However, tacrine was withdrawn from the market in 2012 due to its poor bioavailability and side effects (especially hepatotoxicity) [26]. Despite the small number of commercially available ChE inhibitors, this group of drugs remains the basis for symptomatic treatment of several diseases associated with dysfunction of the cholinergic system (e.g., Alzheimer’s disease, myasthenia gravis, glaucoma, dementia associated with Parkinson’s disease, and traumatic brain injury) [26,27,28].

Studies of Alzheimer’s disease show that the expression and function of both ChE enzymes are altered in the early stages of Alzheimer’s disease and during its progression. The expression of AChE is increased in the early stages of the disease. As the disease progresses, AChE expression decreases to only 33% to 45% of normal levels, while BChE expression in certain brain regions is increased by 40% to 90% of normal levels [29]. People with Alzheimer’s disease suffer from an acetylcholine deficit due to altered ChE function and a loss of cholinergic neurons, resulting in a loss of cognitive functions [30,31]. The primary drug-of-choice for symptomatic treatment of Alzheimer’s disease are ChE inhibitors, which prolong the lifetime of acetylcholine in synapses, improve cognitive function, and limit the clinical signs of Alzheimer’s disease [32]. Considering the mentioned findings, it is crucial to develop new compounds that can inhibit both enzymes (AChE and BChE) simultaneously, or selectively inhibit only BChE [33].

However, in addition to therapeutic effects, this group of drugs may also have several adverse effects due to the inhibition of ChE enzymes in non-target tissues, including activation of muscarinic acetylcholine receptors (mAChR) in the central nervous system (tremor, bradycardia), mixed central and peripheral mAChR activation (nausea, vomiting), peripheral stimulation of mAChR (diarrhea), and overstimulation of nicotinic acetylcholine receptors (nAChRs) in the neuromuscular junction (muscle weakness) [34,35]. It is also known that some compounds with an inhibitory effect on ChEs can antagonize nAChRs in the central and peripheral neuromuscular system and cause paralysis of skeletal muscle fibers [36,37].

In Alzheimer’s disease, BChE expression and function are increased in certain brain regions [29]. Based on this, in our study we focused on the development of new organoruthenium(II) complexes that are selective and potent inhibitors of BChE, and one of them was novel complex **C1**. Our aims were to: (i) develop and biochemically characterize a new organoruthenium(II) complex as a selective BChE inhibitor, and (ii) exclude the effects of this inhibitor on neuromuscular transmission.

## 2. Results and Discussion

### 2.1. Synthesis of **C1**

**Scheme 1:**
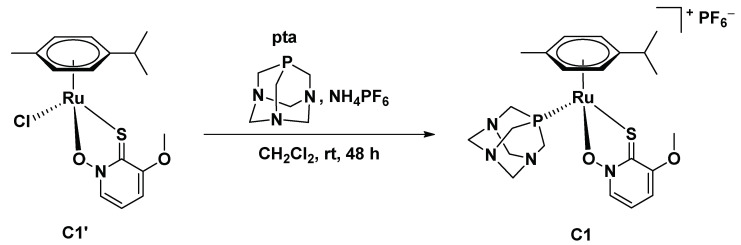
Synthesis pathway of the complex **C1**.

Organoruthenium(II) complex **C1** was prepared from its chlorido analogue **C1′** (Scheme 1). The complex **C1′** was dissolved in dichloromethane, to which the salt NH_4_PF_6_ and the phosphine ligand **pta** were added. The salt NH_4_PF_6_ played a role in the deprotonation of the chloride ion from the **C1** complex since the chlorido ligand itself does not dissociate from the ruthenium atom. White solid NH_4_Cl precipitated as a by-product. After dissociation of the chloride, the **pta** ligand bound to a vacant site via the phosphorus atom, while PF_6_^−^ aimed for the electroneutrality of the organoruthenium(II) complex **C1** in an uncoordinated manner. The reaction mixture was stirred in the dark for 48 h because of the photosensitivity of the phosphine ligand. The unreacted phosphine ligand **pta**, which was added in excess, was filtered off together with NH_4_Cl over Celite. The resulting clear orange mother liquor containing complex **C1** was concentrated under reduced pressure to afford an oily residue to which diethyl ether was added. Complex **C1** precipitated as an orange solid, which was filtered off and dried at 45 °C.

The final compound **C1** as well as the ligand **L1** and the precursor **C1′** were well-characterized by ^1^H and ^31^P NMR spectroscopy (Figures S1–S4), elemental analysis, high-resolution mass spectrometry (HRMS), UV-vis, and IR spectroscopy (Figures S5 and S6). In the ^1^H NMR spectrum of **C1**, there were resonances for three protons of the ligand **L1** in the aromatic region between 7.06 and 7.95 ppm, whereas signals for the aromatic protons of *p*-cymene ring were found in the range between 5.85 and 6.26 ppm. Further, twelve peaks for phosphine ligand **pta** occurred at 4.10–4.48 ppm. Other signals of aliphatic protons can be found under 4.03 ppm, starting with a singlet signal for methoxy protons of the ligand **L1**, and a further heptet, singlet, and two doublets occur at 2.27, 2.15, 1.28, and 1.25 ppm, respectively, for non-aromatic *p*-cymene protons. The ^31^P NMR spectrum showed two signals for phosphorous atoms, belonging to the **pta** ligand and the uncoordinated PF_6_^−^ counter-ion at −31.64 (singlet) and −144.25 (heptet) ppm, respectively. HRMS confirmed the ion mass without the PF_6_^−^ counter-ion, where the calculated *m/z* for [C_22_H_32_N_4_O_2_PRuS]^+^ was 549.1027 and the experimental value for [M–PF_6_]^+^ was 549.1029. Furthermore, elemental analysis confirmed the purity of the product (ΔC: 0.00, ΔH: 0.08, ΔN: 0.14), which needs to agree to within ±0.4% of the calculated values. In IR spectrum bands for aromatic and alkyl, C–H stretching vibrations can be observed between 3200 and 2800 cm^−1^, whereas at around 1500 and 1400 cm^−1^, C=C aryl and C–H alkyl vibrations occurred, respectively, indicating the presence of *p*-cymene and *O,S*-pyrithione ligand. The band at around 1540 cm^−1^ could belong to the *N*-oxide group of the pyrithione moiety. Fingerprint regions of the compounds represent complicated series of absorptions [38]. There were two maxima in the UV-vis spectrum of **C1**, with the first one belonging to the intra-ligand charge transfer, whereas the second maximum belonged to the charge transfer between the ligand and ruthenium ion.

### 2.2. Inhibition of Cholinesterase Enzymes by **C1**

**Figure 1:**
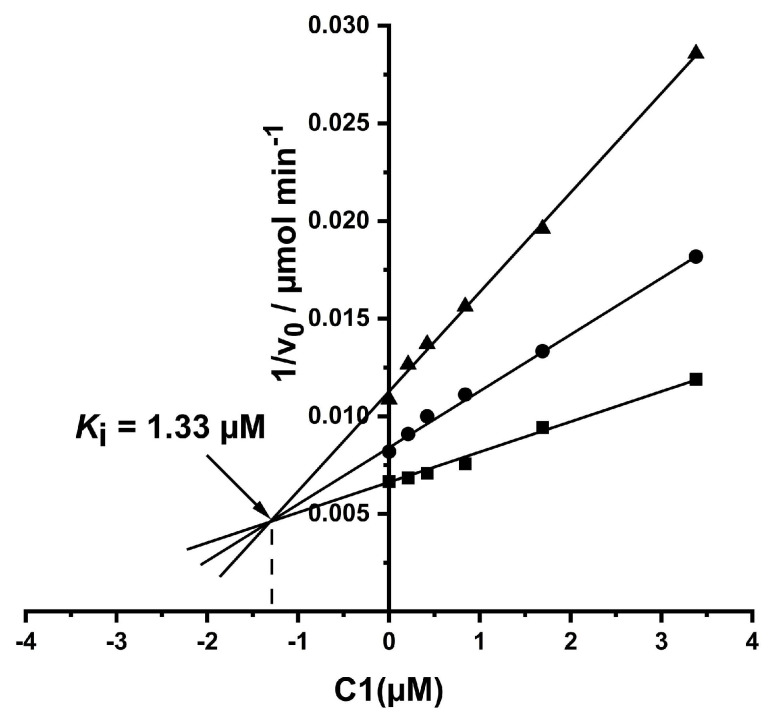
Determination of the type of inhibition and inhibitory constant (*K_i_*) for **C1** towards hsBChE. The concentrations of the substrate acetylthiocholine were 0.125 mM (triangles), 0.25 mM (circles), and 0.5 mM (squares).

In the present study, the newly synthesized complex **C1** was evaluated for its inhibitory activity against AChEs and BChEs of animal and human origin, namely electric eel AChE (eeAChE), human recombinant AChE (hrAChE), horse serum BChE (hsBChE), human recombinant BChE (hrBChE), and canine serum BChE (csBChE). The inhibition parameters for this compound toward ChEs (i.e., IC_50_, *K_i_*) are shown in Table 1. The selected complex did not inhibit eeAChE or hrAChE, but it selectively inhibited hsBChE. The inhibitory potential of **C1** toward hsBChE was within a pharmaceutically interesting range, with an IC_50_ value of 2.88 µM. This inhibition was apparently of a reversible competitive type, indicating that **C1** interacts with the active site within the enzyme gorge (Figure 1). The chlorido analogue **C1′** non-selectively inhibited all tested enzymes (eeAChE, hrAChE, hsBChE, hrBChE, and csBChE) in the low micromolar range (Table 1). These inhibitions were all of the reversible competitive type, indicating that **C1′** interacts with the active site within the enzyme gorge. If we compare complex **C1** with its chlorido analogue **C1′**, we can see that the selectivity of the complex against BChE derived from the addition of the **pta** ligand. In contrast, the inhibitory activity of both compounds toward hsBChE remained the same. Ligand **L1** (1-hydroxy-3-methoxypyridine-2(1*H*)-thion) did not inhibit the activity of the assayed enzymes, while measurement of the inhibitory activity of ligand **pta** was not possible due to solution staining upon contact of **pta** with the substrate (Table 1). In our previous study [9], we also evaluated the inhibitory activity of the ruthenium precursor **P1** (dichloro(*p*-cymene)ruthenium(II) dimer), which only inhibited hsBChE, with the IC_50_ value of 32.7 µM (Table 1).

During the initial screening of anti-ChE activity, hsBChE was only included in the study as a model BChE enzyme. Based on literature findings, hsBChE possesses 90% amino acid sequence identity, similar biochemical and biophysical properties, and structural similarities with the human enzyme. It can therefore be considered as a good model of the human enzyme for toxicological and pharmacological testing, especially because of its commercial availability [39]. However, our further experiments with **C1** using non-commercially available human and canine BChEs did not reproduce the levels of inhibition obtained with BChE derived from horse serum. The complex did not inhibit csBChE at all in the concentrations (0–180 µM) tested, and the inhibition of hrBChE (IC_50_ = 144.2 µM) was about 50 times lower compared to hsBChE. The most likely reason for this difference lays in the primary structures of horse and human BChEs, that differ in fifteen amino acid residues [40,41]. Differences in residues at positions 69 (close to the peripheral binding site of BChE), 277, and 285 (active site gorge of BChE) are most likely responsible for the difference in inhibition of hsBChE and human BChE. Compared to hsBChE, in human BChE, at position 69 threonine residue is replaced by isoleucine, at position 277 valine residue is replaced by alanine, and at position 285 leucine residue is replaced by proline [42]. To further clarify the mechanism of BChE’s interaction with **C1**, molecular docking would have to be used in the future. Indeed, as can be seen from our results, testing ChE inhibitors on hsBChE does not always necessarily provide a reliable basis for toxicological and pharmacological studies in humans.

### 2.3. Effect of **C1** on Skeletal Muscle Contraction In Vitro

The use of ChE inhibitors can have several previously described adverse effects and may also affect skeletal muscles, resulting in uncontrolled muscle contractions and disruption of neuromuscular transmission [34,35]. For this reason, physiological evaluation of potent ChE inhibitors on isolated skeletal neuromuscular preparations is a very important part of preclinical testing. In our case, muscle contraction and membrane potentials were recorded on an isolated mouse hemidiaphragm.

**Figure 2:**
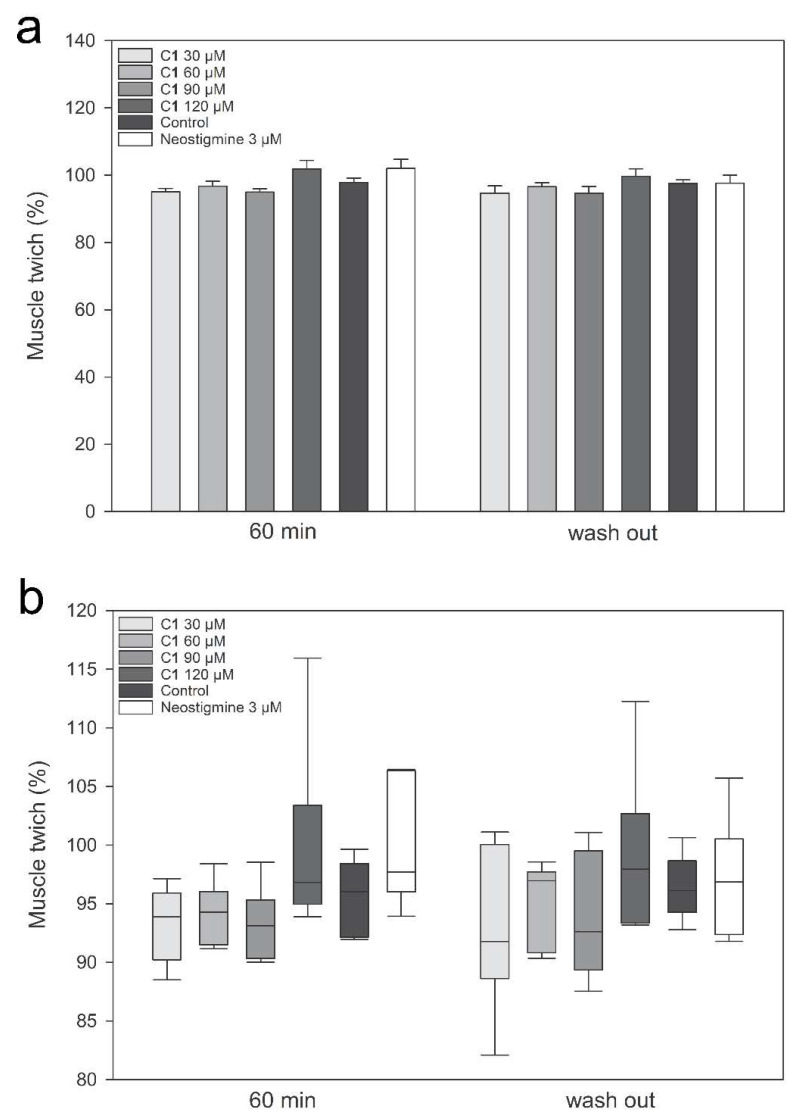
Effects of **C1** on nerve-evoked and directly muscle-elicited single twitch of isolated mouse nerve-hemidiaphragm preparation. The effects of increasing concentrations of **C1** (30, 60, 90, and 120 µM), 0.104% (*v*/*v*) DMSO (control), and 3 µM neostigmine (positive control) on nerve-evoked and directly muscle-elicited single twitch of isolated mouse nerve-hemidiaphragm preparation after 60 min of exposure and after wash-out. (**a**) Values of nerve-evoked single twitch are expressed as mean ± SE from 6–7 different hemidiaphragms. (**b**) Box plots indicate the median values of the directly muscle-elicited single twitch obtained from 6–7 different hemidiaphragms, as well as the interquartile range, with whiskers showing the entire range of data, and all outliers.

**Figure 3:**
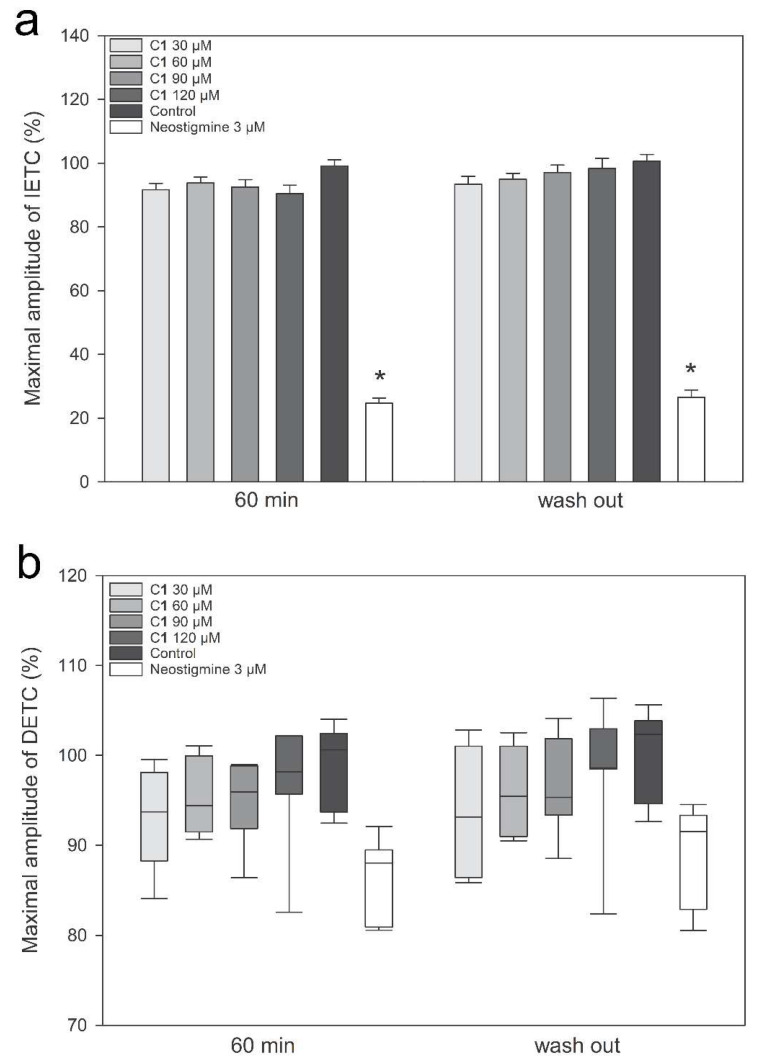
Effects of **C1** on nerve-evoked and directly muscle-elicited tetanic contraction of isolated mouse nerve-hemidiaphragm preparation. The effects of increasing concentrations of **C1** (30, 60, 90 µM, and 120 µM), 0.104% (*v*/*v*) DMSO (control), and 3 µM neostigmine (positive control) on nerve-evoked and directly muscle-elicited tetanic contraction of isolated mouse nerve-hemidiaphragm preparation after 60 min of exposure and after wash-out. (**a**) Values of nerve-evoked tetanic contraction are expressed as mean ± SE from 6–7 different hemidiaphragms. (**b**) Box plots indicate the median values of the directly muscle-elicited tetanic contraction obtained from 6–7 different hemidiaphragms, as well as the interquartile range, with whiskers showing the entire range of data, and all outliers. * *p* ≤ 0.05 versus control (0.104% (*v*/*v*) DMSO).

Biochemical characterization of **C1** indicated that the concentration of 3 µM is the approximate IC_50_ value for hsBChE. For studying the adverse effects of **C1** on mouse neuromuscular preparations, concentrations corresponding to 10-, 20-, 30-, and 40-fold of the IC_50_ value for hsBChE (30, 60, 90, and 120 µM) were used. The effects of different **C1** concentrations on neuromuscular transmission, nerve-evoked, and directly elicited single-twitch and tetanic contractions on hemidiaphragm preparations were studied. A well-known reversible ChE inhibitor, neostigmine, was used as a positive control at a 3 µM concentration, at which it inhibits AChE in mouse diaphragm muscle by 96% [22] and induces characteristic facilitation of neuromuscular transmission [43]. None of the tested concentrations of **C1** had any significant effect on the maximal amplitude of nerve-evoked and directly elicited single-twitch and tetanic contractions (Figure 2a,b and Figure 3a,b). At the highest **C1** concentration tested (120 µM), not statistically significant increases in nerve-evoked single-twitch and tetanic contractions were observed, respectively (Figure 2a and Figure 3a). Inhibition of ChEs (especially AChE) in the neuromuscular junction is associated with the inability to sustain a tetanic contraction due to depolarizing postsynaptic block produced by repetitive high-frequency stimulation of the motor nerve. When AChE is inhibited in the motor end-plate, a slight increase in the amplitude of the nerve-evoked single-muscle contraction and a tetanic fade would be observed, as with neostigmine (Figure 2a and Figure 3a) [43]. At other **C1** concentrations, we did not observe any characteristic features that would indicate nAChR inhibition (decreased amplitude of nerve-evoked single and tetanic contractions). In addition, we also did not detect an increase in the resting muscle tension, which would indicate a possible myotoxic effect of the **C1** [44]. The skeletal muscle contraction results suggest that complex **C1**, even at the highest concentration, has no effect on AChE and most likely no effect on nAChR at the neuromuscular junction.

### 2.4. Effects of **C1** on Membrane Potentials

To exclude the adverse effects of the highest **C1** concentration (120 µM) used on neuromuscular transmission and resting muscle fibers’ potential of mouse hemidiaphragm muscle fibers, we performed measurements of resting membrane potential (rVm), miniature end-plate potentials (MEPPs), and end-plate potentials (EPPs).

**Figure 4:**
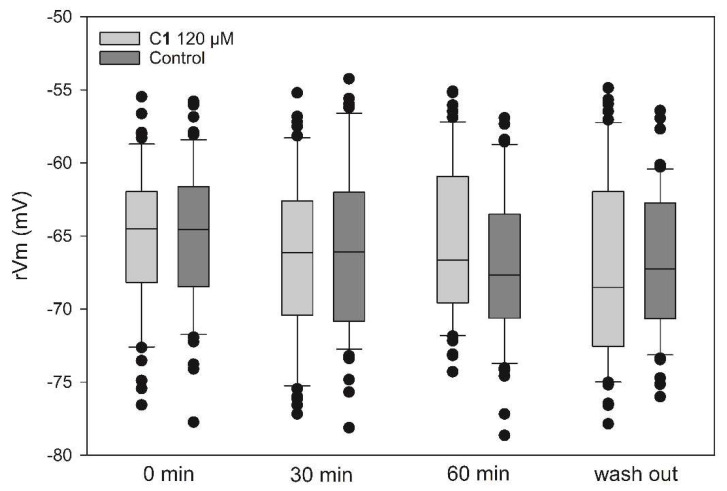
Time-dependent effect of 120 μM of **C1** on the resting membrane potential (rVm) of mouse hemidiaphragm fibers. The neuromuscular preparations were exposed to a 0.104% (*v*/*v*) DMSO concentration and the highest **C1** concentration. Measurements were performed before (time 0) and 30 and 60 min after application of the highest **C1** concentration or the 0.104% (*v*/*v*) DMSO concentration (control), and after washing the preparation with standard Krebs-Ringer physiological solution for about 15 min. Box plots indicate the median values of the data obtained from 4 different hemidiaphragms (n = 7–10 fibers from each), as well as the interquartile range, with whiskers showing the entire range of data, and all outliers.

**C1** at a concentration of 120 µM had no effect on rVm recorded in the end-plate regions of surface muscle fibers after 30 and 60 min of exposure (Figure 4). The median values of rVm were −64.49 mV (IQR: −68.19 to −61.96) under control conditions (i.e., at time zero, immediately before **C1** application), −66.13 mV (IQR: −70.44 to −62.60), and −66.63 mV (IQR: −69.57 to −60.92), 30 and 60 min after exposure to 120 μM of **C1**. These results indicate that **C1** has no myotoxic or depolarizing effect on skeletal muscle fibers.

**Figure 5:**
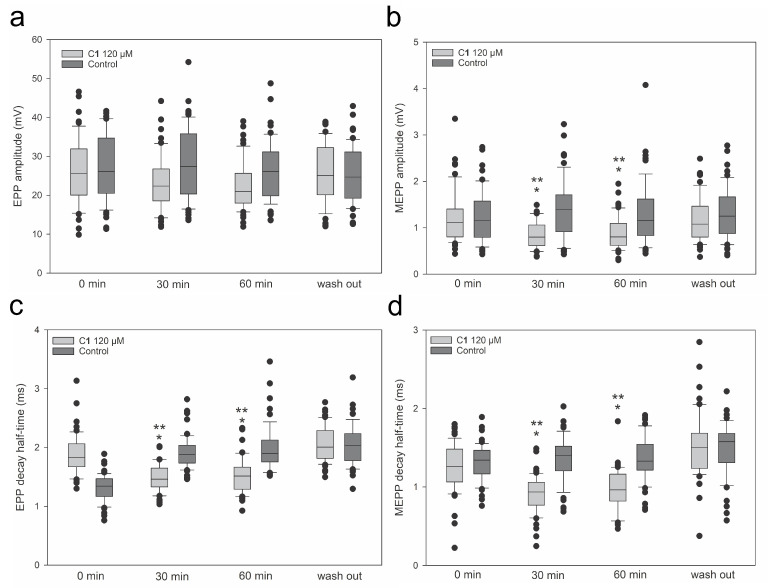
Time-dependent effect of **C1** at 120 μM on the amplitude of end-plate potential, amplitude of miniature end-plate potential, decay half-time of end-plate potential, and decay half-time of miniature end-plate potential of isolated mouse hemidiaphragm fibers. (**a**) Amplitude of end-plate potential, (**b**) amplitude of miniature end-plate potential, (**c**) decay half-time of end-plate potential, and (**d**) decay half-time of miniature end-plate potential. The neuromuscular preparations were exposed to a 0.104% (*v*/*v*) DMSO concentration (control) and to the highest **C1** concentration (120 μM). Measurements were performed before (time 0) and 30 and 60 min after application of the highest **C1** concentration or 0.104% (*v*/*v*) DMSO (control), and after washing the preparation with standard Krebs-Ringer physiological solution for about 15 min. Box plots indicate the median values of the data obtained from 4 different hemidiaphragms (n = 7–10 fibers from each), as well as the interquartile range, with whiskers showing the entire range of data, and all outliers. * *p* ≤ 0.05 versus control (0.104% (*v*/*v*) DMSO); ** *p* ≤ 0.05 versus time 0 (before applicaton of the complex).

For a more mechanistic investigation of the potential effect of a concentration of 120 µM on BChE, AchE, or nAChR in the motor plate, we performed measurements of the amplitudes and half-decay times of MEPPs and EPPs. Electrophysiological recordings from the superficial fibers of the mouse hemidiaphragm exposed to 120 μM of **C1** for 60 min showed that **C1** significantly decreased the amplitudes of MEPPs within 60 min (Figure 5b) without reducing the MEPPs frequency, but did not affect the amplitudes of EPPs (Figure 5a) within 60 min. In addition, a shortened half-decay time of MEPPs and EPPs (Figure 5c,d) was observed at the 120 μM concentration of **C1** within 60 min of exposure. The decrease of MEPPs amplitude and the shortened half-decay time of MEPPs and EPPs suggest a possible weak inhibitory effect of the complex on muscle-type nAChRs [45]. We did not observe characteristic changes for BchE inhibition in the neuromuscular junction (e.g., decreased frequency of MEPPs and giant MEPPs) [22]. This suggests that **C1** does not inhibit BchE in the neuromuscular junction or that this effect is masked by an effect of **C1** on nAChRs. The integrated results of measurements of muscle contraction parameters and parameters of membrane potentials show that the supra-pharmacological concentration of **C1** (120 µM) does not importantly affect the physiology of neuromuscular transmission and skeletal muscle fiber contraction.

## 3. Materials and Methods

### 3.1. Compound Characterization

All reagents for the synthesis were purchased from commercial suppliers (Fluorochem, Glossop, UK; Strem Chemicals, Newburyport, MA, USA) and used as received. Phosphine ligand **pta** was prepared according to the published procedure. Solvents used for the reactions of the complexes were dried over sodium sulphate, whereas solvents used for the isolation of the compounds were used without further purification or drying. Pre-coated TLC sheets (ALUGRAM^®^ SIL G/UV254 (Macherey-Nagel, Düren, Germany)) were used for following the progress of the reactions and were visualized under UV light. Column chromatography was performed with silica gel 60 (35–70 µm; Merck, Darmstadt, Germany) as a stationary phase. NMR spectroscopy was performed using a Bruker Avance III 500 spectrometer (Bruker BioSpin GmbH, Rheinstetten, Germany) at room temperature. ^1^H NMR spectra were recorded at 500 MHz. Chemical shifts are referenced to deuterated solvent residual peaks CDCl_3_ and (CD_3_)_2_CO at 7.26 and 2.05 ppm (referenced against the central line of the quintet), respectively. ^31^P spectra were recorded at 202 MHz and chemical shifts are reported relative to external standards. The splitting of proton resonances is defined as: s = singlet, d = doublet, t = triplet, q = quartet, hept = heptet, m = multiplet, and br = broad signal. Chemical shift (*δ*) and coupling constants (*J*) are given in ppm and Hz, respectively. Infrared spectra were recorded with a Bruker FTIR Alpha Platinum ATR spectrometer (Bruker, Billerica, MA, USA). High-resolution mass spectra (HRMS) were recorded on an Agilent 6224 Accurate Mass TOF LC/MS instrument (Agilent Technologies, Santa Clara, CA, USA). Elemental analyses were carried out on a Perkin–Elmer 2400 II instrument (CHN; Perkin-Elmer, Waltham, MA, USA). UV-Vis spectra for compounds were collected on a Perkin–Elmer LAMBDA 750 UV/Vis/near-IR spectrophotometer (Perkin-Elmer, Waltham, MA, USA).

### 3.2. Synthesis

Ligand 1-hydroxy-3-methoxypyridine-2(1*H*)-thion (**L1**), its chlorido **C1′**, and **pta** complex **C1** were prepared according to the reported protocol in [46].

1-hydroxy-3-methoxypyridine-2(1*H*)-thion (c). Yellow solid, η = 86%. NMR: ^1^H NMR (500 MHz, CDCl_3_): *δ* = 12.33 (bs, 1H, N–O*H*), 7.85 (dd, 1H, *J* = 6.6, 1.3 Hz, Ar–*H*), 6.80–6.71 (m, 2H, Ar–*H*), 3.96 (s, 3H, Ar–OC*H*_3_) ppm. ESI-HRMS: calcd *m/z* [C_6_H_8_NO_2_S]^+^ 158.0270; found [M + H]^+^ 158.0269. Elemental analysis: calcd (%) for C_6_H_7_NO_2_S: C, 45.85; H, 4.49; N, 8.91; found: C, 45.78; H, 4.53; N, 8.95.

[(η^6^-*p*-Cymene)Ru(II)(1-hydroxy-3-methoxypyridine-2(1*H*)-thionato)Cl] (**C1′**). Red solid, η = 71%. NMR: ^1^H NMR (500 MHz, CDCl_3_): *δ* = 7.78 (d, 1H, *J* = 6.8 Hz, Ar–*H* c), 6.71 (dd, 1H, *J* = 7.9, 6.8 Hz, Ar–*H* c), 6.54 (d, 1H, *J* = 7.9 Hz, Ar–*H* c), 5.48 (d, 2H, *J* = 5.9 Hz, Ar–*H* cymene), 5.26 (d, 2H, *J* = 5.9 Hz, Ar–*H* cymene), 3.88 (s, 3H, Ar–OC*H*_3_ c), 2.83 (hept, 1H, *J* = 6.9 Hz, Ar–C*H*(CH_3_)_2_ cymene), 2.23 (s, 3H, Ar–C*H*_3_ cymene), 1.26 (d, 6H, *J* = 6.9 Hz, Ar–CH(C*H*_3_)_2_ cymene) ppm. ESI-HRMS: calcd *m/z* [C_19_H_20_NORuS]^+^ 392.0258; found [M − Cl]^+^ 392.0250. Elemental analysis: calcd (%) for C_16_H_20_ClNO_2_RuS: C, 45.01; H, 4.72; N, 3.28; found: C, 45.16; H, 4.67; N, 3.25. IR: ṽ (cm^−1^, ATR, selected peaks): 2962, 1548, 1449, 1425, 1418, 1252, 1067, 767, 670. UV-Vis: λ (nm) (ε (L mol^−1^ cm^−1^); c = 5 × 10^−5^ M, MeOH): 273 (14906), 337 (5717), 480 (686).

[(η^6^-*p*-Cymene)Ru(II)(1-hydroxy-3-methoxypyridine-2(1*H*)-thionato)pta]PF_6_ (**C1**). Yellow solid, η = 77%. NMR: ^1^H NMR (500 MHz, (CD_3_)_2_CO)): *δ* = 7.95 (dd, 1H, *J* = 6.6, 1.2 Hz, Ar–*H* c), 7.14–7.06 (m, 2H, Ar–*H* c), 6.26 (d, 1H, *J* = 6.1 Hz, Ar–*H* cymene), 6.18 (d, 1H, *J* = 6.1 Hz, Ar–*H* cymene), 6.02 (d, 1H, *J* = 6.1 Hz, Ar–*H* cymene), 5.85 (d, 1H, *J* = 6.1 Hz, Ar–*H* cymene), 4.48 (s, 6H, *H* pta), 4.22 (dd, 3H, *J* = 14.9, 3.6 Hz, *H* pta), 4.10 (dd, 3H, *J* = 14.9, 3.6 Hz, *H* pta), 4.03 (s, 3H, Ar–*OCH*_3_ c), 2.72 (hept, 1H, *J* = 6.9 Hz, Ar–C*H*(CH_3_)_2_ cymene), 2.15 (s, 3H, Ar–*CH*_3_ cymene), 1.28 (d, 3H, *J* = 6.9 Hz, Ar–CH(*CH*_3_)_2_ cymene), 1.25 (d, 3H, *J* = 6.9 Hz, Ar–CH(C*H*_3_)_2_ cimen) ppm. ^31^P NMR (202 MHz, (CD_3_)_2_CO)): *δ* = −31.68 (*P*–pta), –144.25 (hept, *J*_PF_ = 708 Hz, PF_6_) ppm. ESI-HRMS: calcd *m/z* [C_22_H_32_N_4_O_2_PRuS]^+^ 549.1027; found [M–PF_6_]^+^ = 549.1029. Elemental analysis: calcd (%) for C_22_H_32_F_6_N_4_O_2_P2RuS: C, 38.10; H, 4.65; N, 8.08; found: C, 38.10; H, 4.73; N, 7.94. IR: ṽ (cm^−1^, ATR, selected peaks): 2929, 1425, 1062, 971, 947, 828, 800, 581, 574, 470. UV-Vis: λ (nm) (ε (L mol^−1^ cm^−1^); c = 5 × 10^−5^ M, MeOH): 278 (14272), 372 (4033).

### 3.3. Cholinesterase Inhibition Assay

Cholinesterase activity was measured by Ellman’s method [47] adapted for microtiter plates, as previously described by Ristovski et al. [9]. A complex and its ligands were first screened for the IC_50_ determination and then the inhibitory constants (*K_i_*) were determined. A stock solution of **C1** (1 mg/mL) was prepared in 5% *v*/*v* DMSO in deionized water, while a stock solution of chlorido analogue **C1′** (1 mg/mL) was prepared in 100% methanol (MeOH). Stock solutions of ligands **L1** and **pta** (1 mg/mL) and the positive control (neostigmine methyl sulphate, 1 mg/mL, Tokyo Chemical Industry Co., Ltd., Tokyo, Japan) were prepared in 100% MeOH. These solutions were added to the wells, and gradually diluted in 100 mM of potassium phosphate buffer (pH 7.4) to the final volume of 50 µL. Acetylthiocholine chloride and 5,5′-dithiobis-2-nitrobenzoic acid were then dissolved in the same buffer to the respective final concentrations of 1 and 0.5 mM and added (100 μL) to the wells of the microtiter plates. **C1** was screened against a suite of ChEs of human and animal origin (eeAChE, hrAChE, hsBChE) (all three from Sigma-Aldrich, St. Louis, MO, USA), hrBChE (gift from the research group of Professor Stanislav Gobec, Faculty of Pharmacy, University of Ljubljana), and csBChE. All enzymes were dissolved in the 100 mM potassium phosphate buffer (pH 7.4) to 0.0075 U/mL. Fifty μL of each ChE was added to start the reactions, which were followed spectrophotometrically at 405 nm and 25 °C for 5 min using a kinetic microplate reader (Dynex Technologies Inc., Chantilly, VA, USA). The blank reactions without the inhibitors were run with the appropriate dilutions of the solvents, in which the tested compound and positive control were initially diluted (5% aqueous DMSO or 100% MeOH), and the readings were corrected according to the appropriate blanks. At the end of the experiments, the concentrations of compounds causing 50% inhibition of ChE activity (IC_50_) were determined. To determine **C1** inhibition constants (*K_i_*), the kinetics were monitored using three different final substrate concentrations (0.125, 0.25, 0.5 mM). Each measurement was repeated at least three times. Data were analyzed using OriginPro software (OriginPro 2020, OriginLab Corporation, Northampton, MA, USA).

### 3.4. Experimental Animals and Neuromuscular Preparations

Here, 35 conventional 6–14-week-old male Balb/c mice were originally obtained from Envigo RMS Srl (Udine, Italy) and bred at the Laboratory Animal Breeding and Experimental Facility at Veterinary Faculty, University of Ljubljana. At the time of performing the experiments, all mice were 17–21 weeks old. All mice were housed, five in each cage (1284L EUROSTANDARD TYPE II L, 365 × 207 × 140 mm, floor area 530 cm^2^; Tecniplast, Buguggiate, Italy), under standard conditions (T s = 22–24 °C; RH = 40–60%), on a 12/12 light/dark cycle (lights on at 02:00 a.m.). Food (Teklad global 16% protein extruded, irradiated, 2916; Envigo RMS Srl, Udine, Italy) and acidified tap water with the addition of HCl (pH 3–4) were provided ad libitum. For bedding, autoclaved wood fibers were used (LIGNOCEL 3/4-S; J. Rettenmaier and Söhne GmbH + Co KG, Rosenberg, Germany). The experiments on isolated neuromuscular preparations followed ethical standards, were carried out in strict accordance with the Slovenian legislation, as harmonized with the European Communities Council Guidelines (Directive 86/609/EGS on 24 November 1986; Directive 2010/63/EU on 22 September 2010), and were approved by the Administration of the Republic of Slovenia for Food Safety, Veterinary, and Plant Protection (Permit No. U34401–8/2018/4).

Mice were sacrificed by cervical dislocation and immediate exsanguination. After dissection of the diaphragm muscle with both phrenic nerves, the diaphragm muscle was cut into two hemidiaphragms. Each hemidiaphragm was maintained in oxygenated standard Krebs-Ringer solution (154 mM NaCl, 5 mM KCl, 2 mM CaCl_2_, 1 mM MgCl_2_, 5 mM HEPES, and 11 mM D-glucose; pH 7.4). Just prior to experiments, **C1** was dissolved in a solution of 2.5% DMSO in deionized water at a stock concentration of 2 mg/mL. The final concentration of DMSO in the organ bath was always 0.104% *v*/*v*. All experiments were performed at room temperature (22–24 °C).

### 3.5. Muscle Contraction Recordings

The hemidiaphragm with phrenic nerve was pinned on its lateral side into a silicon-coated organ bath containing oxygenated Krebs-Ringer (K-R) solution. Then, the tendon part was linked to the lever of an isometric mechano-electrical transducer (Grass Instruments, West Warwick, RI, USA) via a stainless-steel hook and silk thread. The equal stimulus protocol, equipment, and software were used as previously described by Ristovski et al. [9]. The concentrations of **C1** studied were 30, 60, 90, and 120 μM and their effect on muscle contraction was continuously measured during 60 min after application. The muscle twitch and tetanic contraction blockade produced by **C1** was shown as the percentage of the initial maximal response. The well-known reversible AChE inhibitor neostigmine methyl sulphate (Tokyo Chemical Industry Co., Ltd., Tokyo, Japan) was used as a positive control, and a corresponding solution of 2.5% DMSO in deionized water as a negative control.

### 3.6. Membrane Potential Recordings

Electrophysiological recordings on hemidiaphragm muscle fibers were performed using conventional microelectrode techniques. For measurements of rVm, EPP, and MEPP, hemidiaphragm preparations were immersed in oxygenated K-R solution containing 1.6 μM of μ-conotoxin GIIIB (Bachem, Bubendorf, Switzerland) and incubated for 30 min. The same stimulus protocol, equipment, and software were used to perform membrane potential recordings, as previously described by Ristovski et al. [9]. Only the highest concentration of **C1** (120 μM) was studied. The recordings were performed before applying **C1**, 30 and 60 min after the compound application, and 15 min after the compound wash-out. MEPP and EPP amplitudes were normalized to a rVm of −70 mV using the formula: Vc = Vo (−70)/rVm, where Vc is the normalized amplitude of MEPPs and EPPs, and Vo is their recorded amplitude.

### 3.7. Data Analysis and Statistics

Data were statistically analyzed using Sigma Plot for Windows version 12.5 (Systat Software Inc., San Jose, CA, USA). Data were first tested for normality (Shapiro–Wilk test) and equal variance (Brown–Forsythe test) to assign them to a parametric or nonparametric analysis. One-way analysis of variance (ANOVA) followed by the Holm–Sidak test for multiple comparisons was performed to compare (normal distribution of the data) the effects of different **C1** concentrations on nerve-evoked muscle twitch and nerve-evoked tetanic contraction amplitude. Parametric data are presented as the mean ± SEM. For nonpaired comparisons between multiple groups, when equal variance was not meet, nonparametric analysis was performed using Kruskal–Wallis one-way ANOVA on ranks, followed by Dunn’s post-hoc test, and the results were presented as box and whisker plots using the median and interquartile range (IQR). A *p*-value ≤ 0.05 was considered statistically significant.

## 4. Conclusions

The novel organoruthenium(II) complex **C1** selectively, competitively, and reversibly inhibited hsBChE in the low micromolar range, but did not inhibit hrBChE and csBChE in the pharmaceutically interesting range, and did not inhibit AChE enzymes. None of the tested concentrations (30, 60, 90, and 120 µM) had any significant effect on the maximal amplitude of nerve-evoked and directly elicited single-twitch and tetanic contractions. At the highest concentration tested (120 µM), **C1** had no effect on rVm recorded in end-plate regions of superficial skeletal muscle fibers, but significantly decreased amplitudes and shortened the half-decay time of MEPPs without decreasing their frequency. Overall, **C1** had no significant effect on the physiology of neuromuscular transmission and contraction of skeletal muscle fibers and did not inhibit BChE in the neuromuscular junction of mouse hemidiaphragm preparation, or this effect was masked by an effect of **C1** on nAChRs. **C1** is likely a highly species-specific inhibitor of hsBChE for potential use as a species-specific drug target. Our cumulative results provide the basis for a structure guided approach to develop more potent and selective, species-specific inhibitors.

## Figures and Tables

**Table 1:** Inhibition of animal and human cholinesterase enzymes by **C1**.

Compound	eeAChE		hrAChE		hsBChE		hrBChE		csBChE	
	IC_50_ (µM)^a^	*K_i_* (µM)	IC_50_ (µM)^a^	*K_i_* (µM)	IC_50_ (µM)^a^	*K_i_* (µM)	IC_50_ (µM)^a^	*K_i_* (µM)	IC_50_ (µM)^a^	*K_i_* (µM)
**C1**	/	n.d.	/	n.d.	2.88	1.33	144.2	n.d.	/	n.d.
**C1′**	7.50	4.80	5.04	5.35	2.34	3.40	1.29	1.25	2.27	3.0
**L1**	/	n.d.	/	n.d.	/	n.d.	/	n.d.	/	n.d.
**pta**	n.d.*	n.d.*	n.d.*	n.d.*	n.d.*	n.d.*	n.d.*	n.d.*	n.d.*	n.d.*
**P1** ^(a)^	/	n.d.	/	n.d.	32.7	23.3	n.d.	n.d.	n.d.	n.d.
Neostigmine	3.9	n.d.	4.8	n.d.	62.8	n.d.	173.5	n.d.	28.4	n.d.

IC_50_ was determined as the concentration of the compound inducing 50% inhibition of the enzyme activity. *K_i_*: inhibition constant, /: no activity, n.d.: not determined, and n.d.* not determined due to color reaction of ligand **L1** with the substrate. eeAChE—electric eel acetylcholinesterase; hrAChE—human recombinant acetylcholinesterase; hsBChE—horse serum butyrylcholinesterase; hrBChE—human recombinant butyrylcholinesterase; csBChE—canine serum butyrylcholinesterase. ^(a)^ Inhibition of eeAChE, hrAChE, and hsBChE by **P1** was previously reported by Ristovski et al. [9].

## Data Availability

All the data generated or analyzed during this study are included in this published article (and its Supplementary Materials).

## Supplementary Materials

The supporting information can be downloaded at: https://www.mdpi.com/article/10.3390/ijms24032681/s1.
