# Supplementary material for: Novel Organoruthenium(II) Complex C1 Selectively Inhibits Butyrylcholinesterase without Side Effects on Neuromuscular Transmission
Source: Int J Mol Sci. 2023 Jan 31;24(3):2681. doi: 10.3390/ijms24032681 (PMC9916964; doi:10.3390/ijms24032681)
Supplement: Supplementary file 1 [file ijms-24-02681-s001.zip › ijms-2134298-supplementary.pdf]

Novel Organoruthenium(II) Complex C1 Selectively Inhibit Butyrylcholinesterase without Side-Effects on Neuromuscular Transmission

Tomaž Trobec [1], Monika C. Žužek [1], Kristina Sepčić [2], Jerneja Kladnik [3], Iztok Turel [3] and Robert Frangež [1,*]

[1] Institute of Preclinical Sciences, Veterinary Faculty, University of Ljubljana, Gerbičeva 60, 1000 Ljubljana, Slovenia
[2] Department of Biology, Biotechnical Faculty, University of Ljubljana, Jamnikarjeva 101, 1000 Ljubljana, Slovenia
[3] Department of Chemistry and Biochemistry, Faculty of Chemistry and Chemical Technology, University of Ljubljana, Večna pot 113, 1000 Ljubljana, Slovenia
* Correspondence: robert.frangez@vf.uni-lj.si; +386-1-4779-131

1. NMR spectra

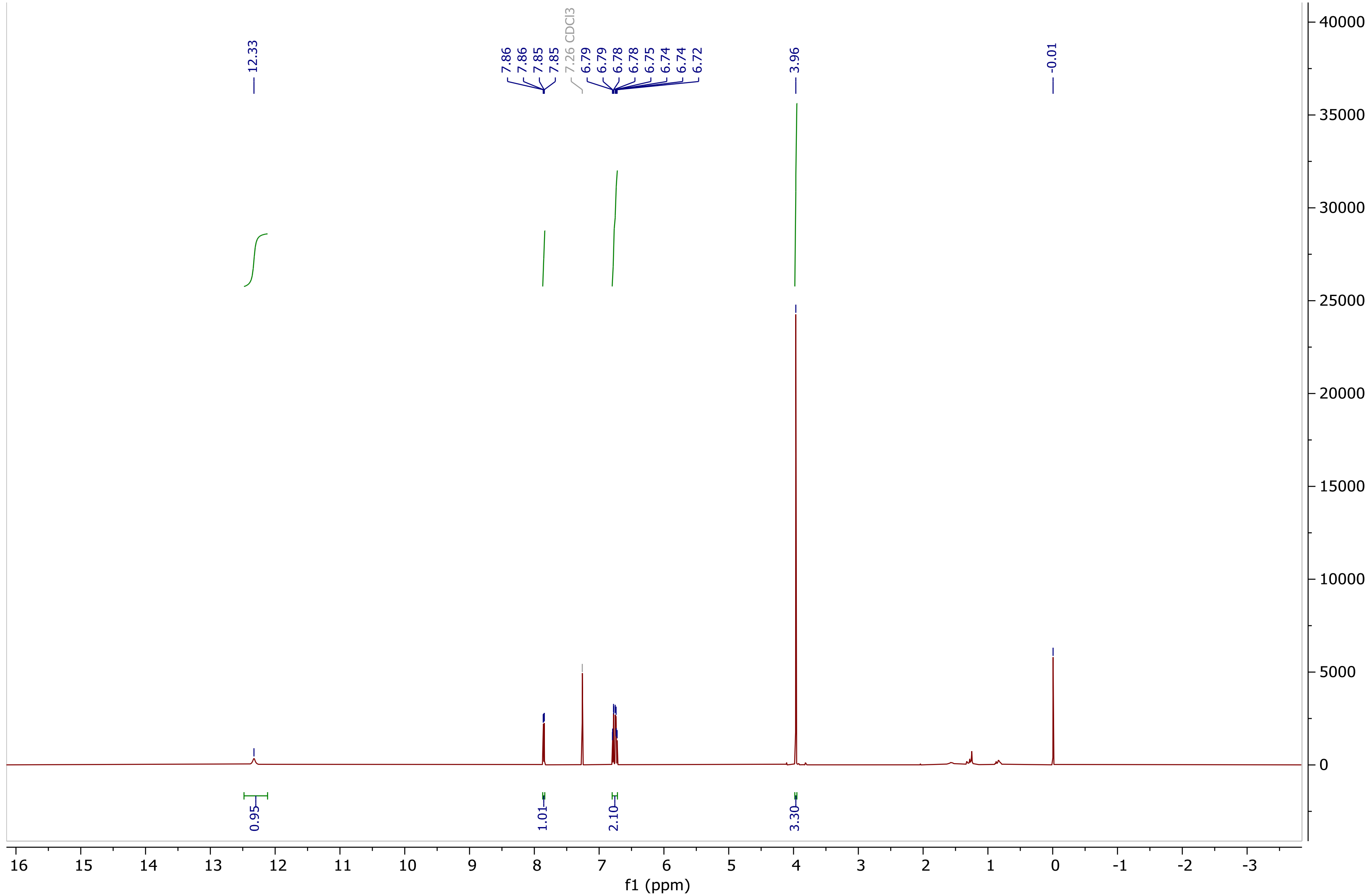

Figure S1: ^{1}H NMR spectrum of the ligand **c** in $CDCl_3$.

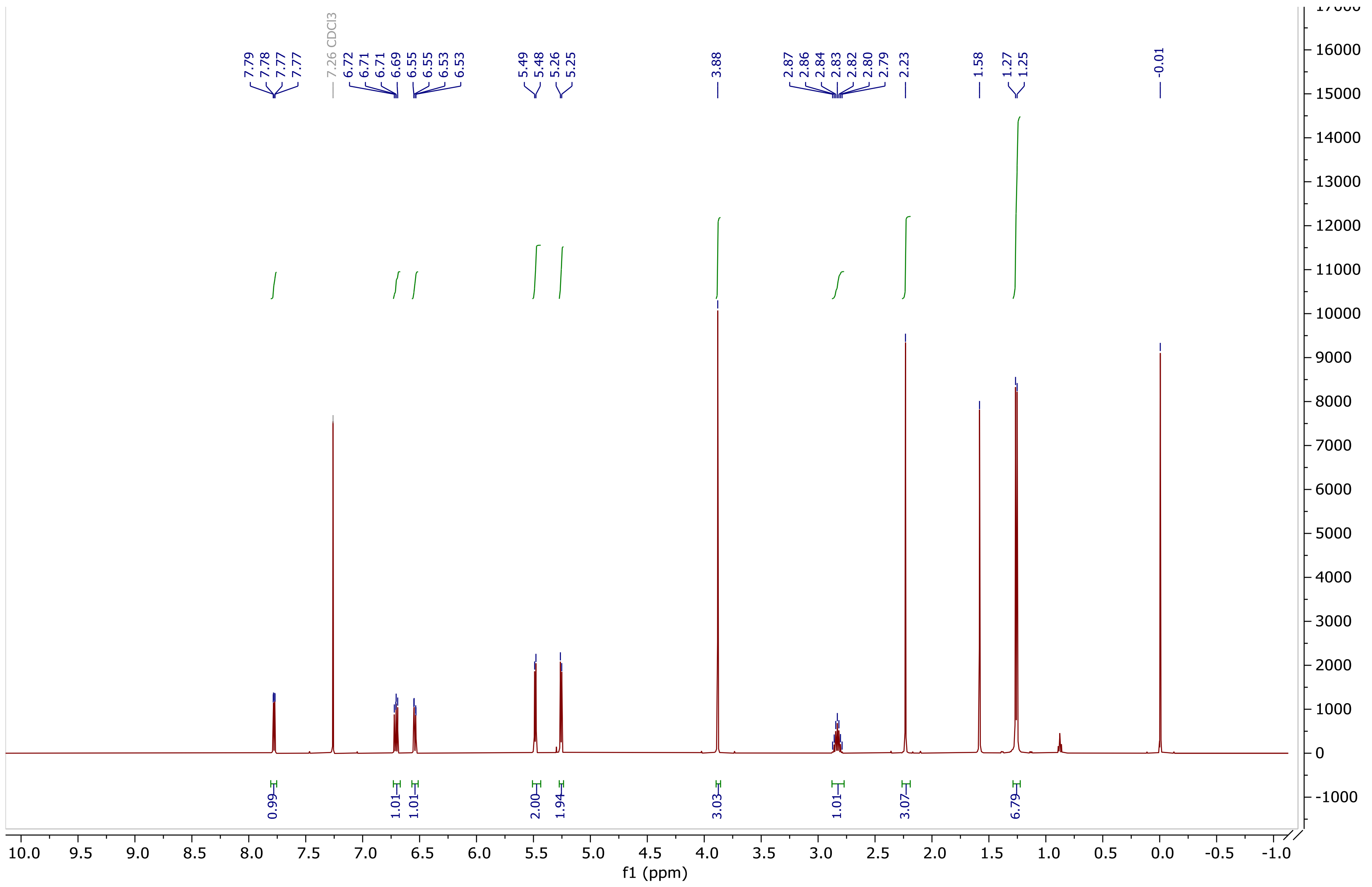

Figure S2: ^{1}H NMR spectrum of the complex **C1'** in $CDCl_3$.

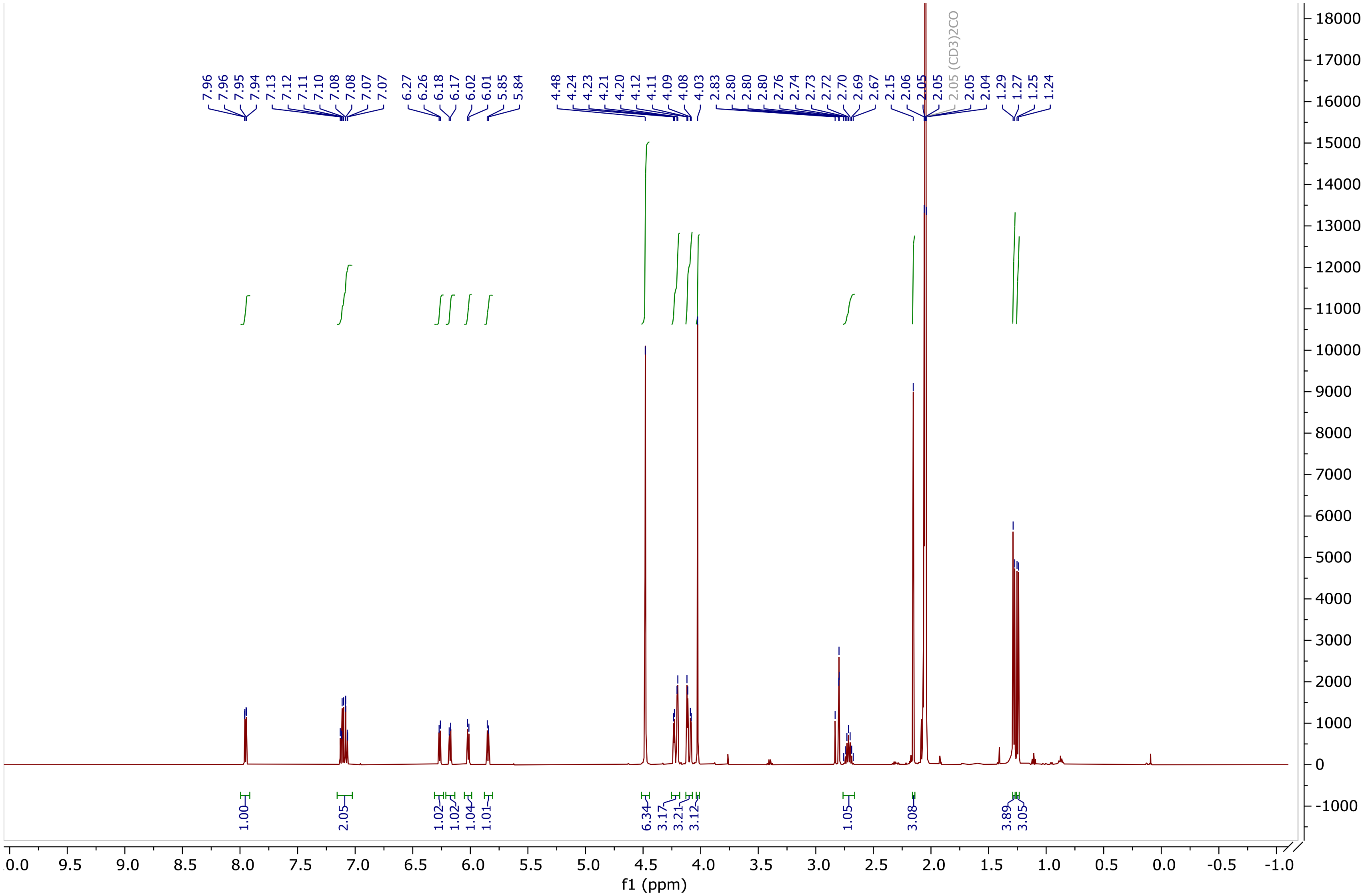

Figure S3: ^{1}H NMR spectrum of the complex **C1** in $(CD)_3CO$.

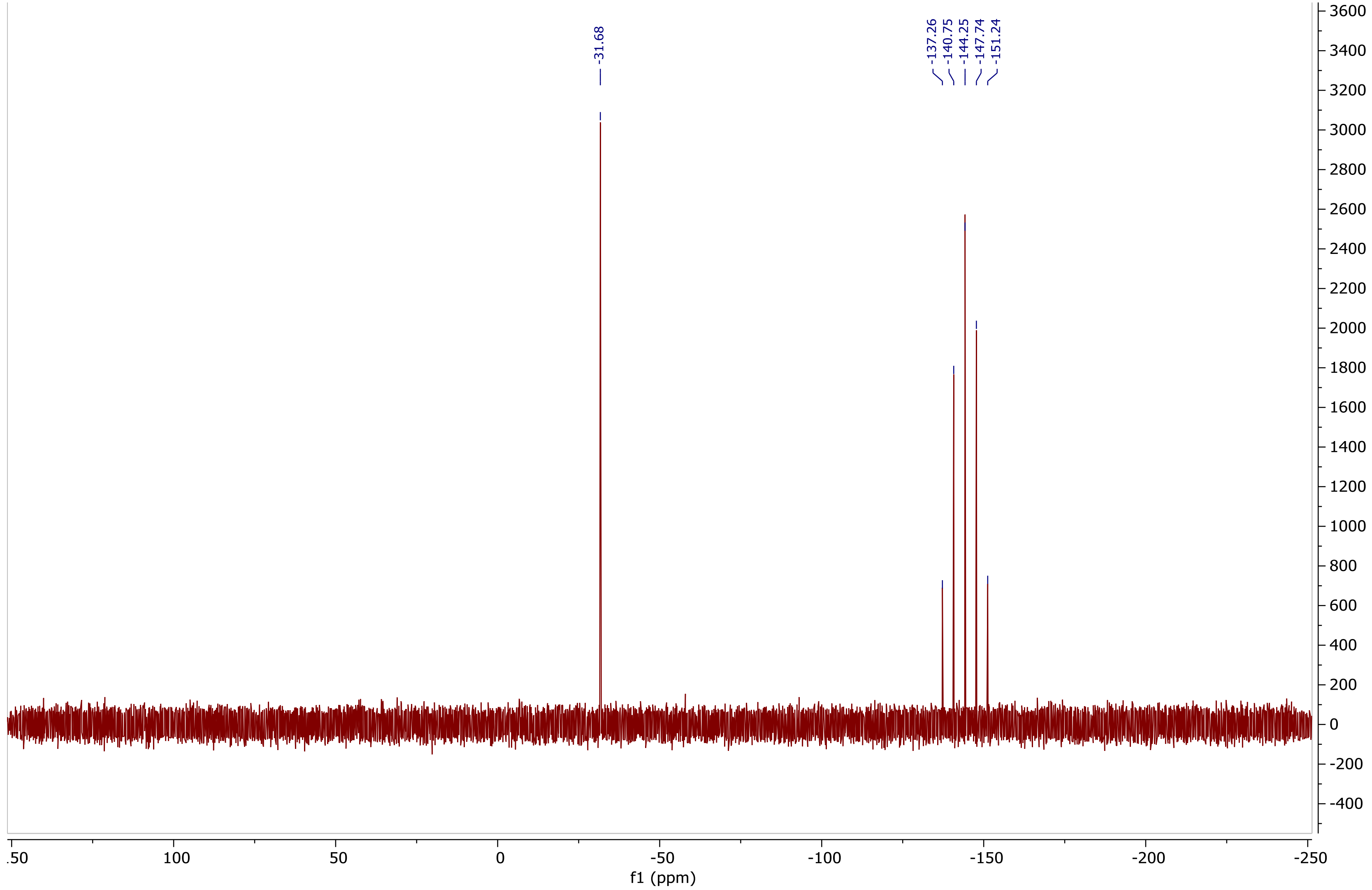

Figure S4: ^{31}P NMR spectrum of the complex **C1** in $(CD)_3CO$.

2. IR spectra

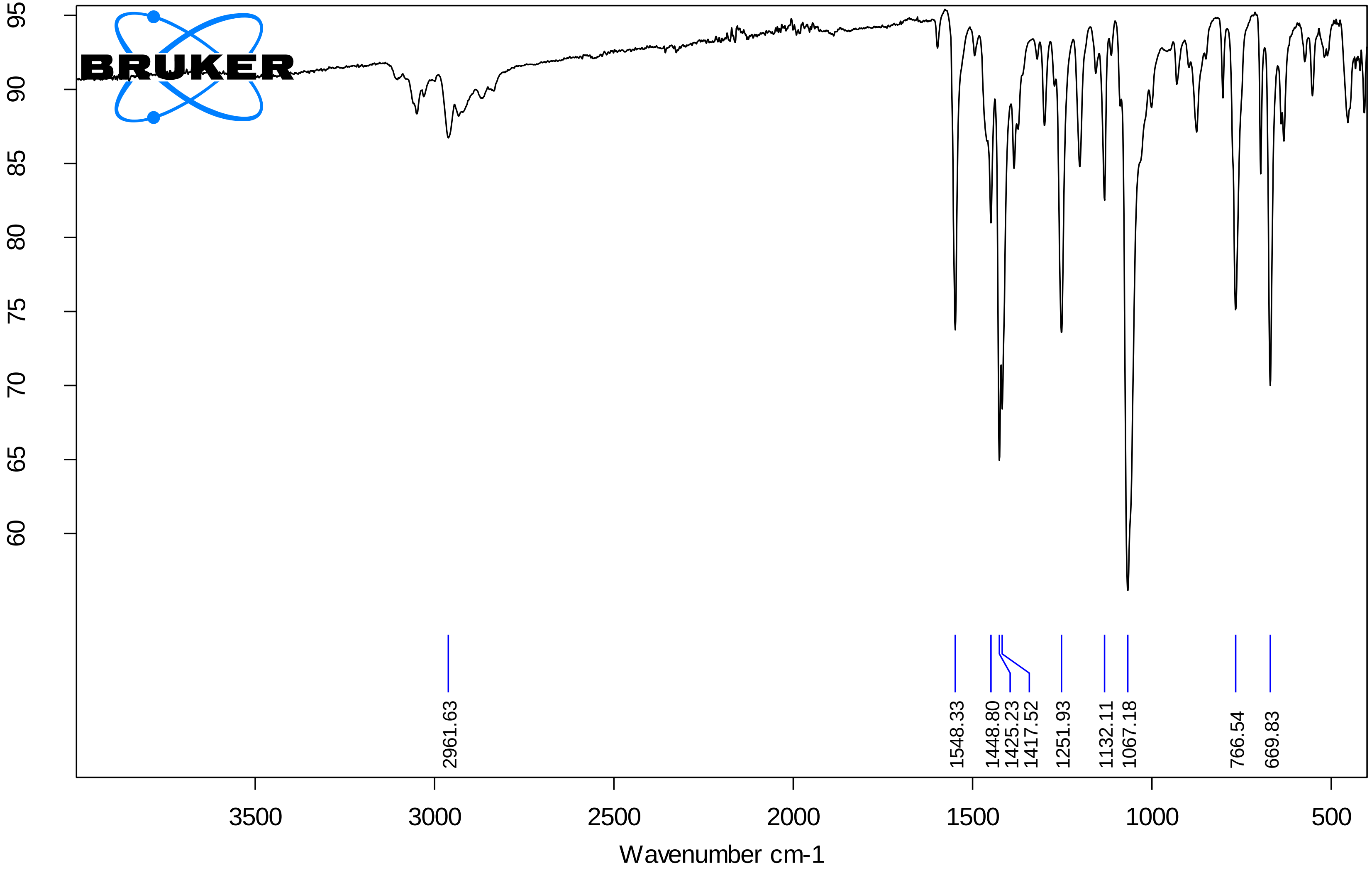

Figure S5: IR spectrum of the complex **C1**.

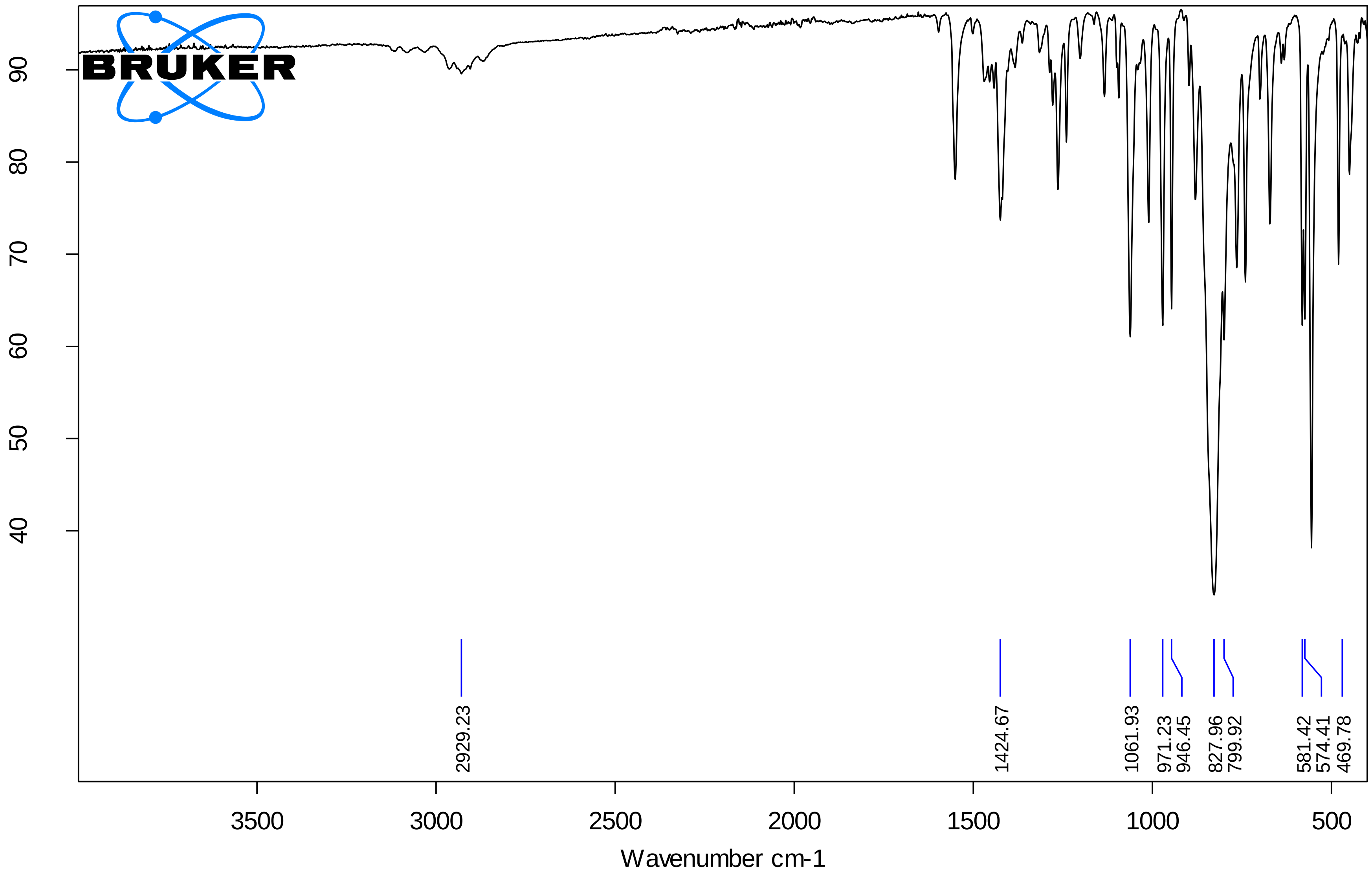

Figure S6: IR spectrum of the complex **C1**.
